# The Role of Antifungals against *Candida* Biofilm in Catheter-Related Candidemia

**DOI:** 10.3390/antibiotics4010001

**Published:** 2014-12-25

**Authors:** Emilio Bouza, Jesús Guinea, María Guembe

**Affiliations:** 1 Department of Clinical Microbiology and Infectious Diseases, Gregorio Marañón Hospital, Madrid 28007, Spain; E-Mails: ebouza@microb.net (E.B.); jguineaortega@yahoo.es (J.G.); 2 Medicine Department, School of Medicine, Universidad Complutense de Madrid, Madrid 28040, Spain; 3 CIBER de Enfermedades Respiratorias (CIBER RES), CB06/06/0058, Madrid, Spain

**Keywords:** biofilm, *Candida*, antifungals, catheter-related candidemia

## Abstract

Catheter-related bloodstream infection (C-RBSI) is one of the most frequent nosocomial infections. It is associated with high rates of morbidity and mortality. *Candida* spp. is the third most common cause of C-RBSI after coagulase-negative staphylococci and *Staphylococcus aureus* and is responsible for approximately 8% of episodes*.* The main cause of catheter-related candidemia is the ability of some *Candida* strains—mainly *C. albicans* and *C. parapsilosis*—to produce biofilms. Many *in vitro* and *in vivo* models have been designed to assess the activity of antifungal drugs against *Candida* biofilms. Echinocandins have proven to be the most active antifungal drugs. Potential options in situations where the catheter cannot be removed include the combination of systemic and lock antifungal therapy. However, well-designed and -executed clinical trials must be performed before firm recommendations can be issued.

## 1. Introduction

*Candida* spp. is the third leading cause of catheter-related infections after coagulase-negative staphylococci and *Staphylococcus aureus*. It is associated with high rates of crude mortality [1,2,3]. *C. albicans* and *C. parapsilosis* are the most frequent fungi in hospitalized patients and in hospital environments [4]. They are also the species that most commonly produce biofilm, a characteristic that facilitates persistent infections, such as catheter-related candidemia (CRC) [5,6,7,8,9,10]. Non-*Candida* biofilms have also been studied, and the lowest metabolic activity was recently reported by Marcos-Zambrano *et al.* [11]. Recent findings also support the hypothesis that surface-associated filamentous fungi can form biofilms [12].

Most studies based on the role of biofilm in infections have been performed with bacteria. Data on the peculiarities of *Candida* biofilm and its clinical significance are scarce and come mainly from studies on the ability of antifungals to eradicate biofilm.

We performed an exhaustive review of *Candida* biofilm and its eradication with antifungals in CRC.

## 2. Catheter-Related Candidemia

Many episodes of candidemia originate in central venous catheters (CVCs) [5,13], leading to high rates of morbidity and mortality [14]. The ability of some *Candida* strains to produce biofilms—mainly *C. albicans* and *C. parapsilosis*—may explain the high frequency of CRC.

Diagnosis of CRC is problematic, owing to the lack of appropriate microbiologic procedures and the difficulty in eradicating Candida biofilms [15,16,17].

## 3. *Candida* Biofilm

### 3.1. Composition

Some bacteria and fungi have the ability to form cell aggregates. In combination with other host components (e.g., fibrin, platelets and immunoglobulins), these aggregates comprise the complex structure of biofilm, which is surrounded by a polymer matrix. Biofilm-producing bacteria or fungi can adhere to natural or artificial surfaces, where the properties they express differ from the properties of their planktonic forms. Consequently, they are more virulent and can cause chronic infections [6,18,19].

### 3.2. Formation

Fungal biofilms are increasingly common as a result of the widespread use of antibiotics, medical devices and the increase in the number of immunocompromised patients [8,20,21]. *Candida* biofilm results from an initial attachment of cells to glycoprotein-coated host cells and tissue or biomaterial surfaces. The second phase (proliferation and biofilm formation) is characterized by the generation of a three-dimensional structure [22,23,24], which is highly dependent on the conditions under which the biofilm is formed (e.g., type of implanted device and its location) [7,18,25].

### 3.3. Genetic Mechanisms of Biofilm Formation

Several molecular factors are associated with biofilm formation. Quorum sensing could play an important role in the dispersal of biofilm cells and the formation of amyloid-dependent adhesion nanodomains and has broad implications as a mechanism underlying yeast cell-cell adhesion and biofilms [26,27,28]. Differential gene expression was recently demonstrated in *Candida* cells under planktonic conditions or as biofilm. The major functional categories of genes upregulated in biofilms are those implicated in transcriptional regulation, protein synthesis, amino acid synthesis, cell wall synthesis, efflux pumps and adhesins [29,30,31].

### 3.4. Measurement of Biofilm Formation

Fungal biofilm formation and eradication of biofilm by antifungals can be assessed using *in vitro* approaches (microtiter plate-based models, flow displacement biofilm models, cell-culture-based models and microfluidic device models) and *in vivo* approaches (CVC models, subcutaneous and intraperitoneal foreign body infection models, urinary tract infection models, ear-nose-throat infection models, respiratory tract infection models and osteomyelitis infection models) [32,33,34,35,36,37,38]. The most common colorimetric assays for measuring biofilm formation and metabolic activity are, respectively, the crystal binding violet assay and the XTT assay (based on 2,3-bis[2-methoxy-4-nitro-5sulfophenyl]-2H-tetrazolium-5-carboxanilide inner salt) [39,40,41,42,43,44,45,46]. However, there are still no standard cut-offs to establish whether a *Candida* strain has high, moderate or low capacity to produce biofilm. Growth of biofilm can also be visualized *in situ* by means of scanning electron microscopy, which is the most commonly used method [33].

### 3.5. Clinical Aspects of Candida Biofilm

The incidence of biofilm-producing *Candida* strains recovered from the blood of candidemic patients was reported to be 83.3% in both the general population and immunocompromised patients [47,48]. Ruiz *et al.* reported a 100% incidence rate of biofilm-producing *C. parapsilosis* strains recovered from children’s blood [49]. These findings have a major clinical impact, because of the increase in antifungal resistance and protection against host defenses. Moreover, fungal biofilm on medical devices, such as CVCs, could act as a reservoir for re-infection and necessitate catheter withdrawal [50,51,52]. Another important factor associated with *Candida* biofilm formation is the administration of lipid emulsion to patients via CVCs, which has been shown to induce germination and enhance biofilm production [53]. Catheter-related bloodstream infections (C-RBSIs) occur in 1.3% to 26.2% of patients with CVCs used to administer parenteral nutrition [54]. However, the role of biofilm production in clinical outcome is still being explored*.*

## 4. Antifungal Activity against *Candida* Biofilm

### 4.1. In Vitro Activity of Antifungals to Eradicate Candida Biofilm

The efficacy of antifungals against *Candida* biofilms has been tested in numerous *in vitro* studies. Amphotericin B (AmB) and echinocandins proved to be the most active agents against *Candida* biofilms. Azoles had little effect against *Candida* biofilms, even at high doses and in combination with caspofungin [55,56,57,58]. However, fluconazole combined with minocycline had a synergistic effect against fluconazole-resistant *C. albicans*, probably owing to the boosting of fluconazole by minocycline as it penetrates biofilm [59].

Most studies evaluating AmB compared it with other families of antifungals. AmB generally has a good anti-biofilm effect at high doses of the liposomal form (AmB-L) [56,60,61]. This observation was also validated by Uppuluri *et al.*, who examined the effect of continuous perfusion with AmB on *C. albicans* biofilms under conditions of flow [57]. Moreover, Tobudic *et al.* evaluated the anti-biofilm effect of AmB in combination with caspofungin and posaconazole against *C. albicans* planktonic and sessile cells and demonstrated that the combination of AmB/posaconazole was synergistic against *C. albicans* biofilms, whereas AmB/caspofungin produced an indifferent interaction [62]. Poorer results were seen when AmB was tested against biofilms of *C. parapsilosis* [55].

Studies evaluating the susceptibility of *Candida* biofilms to echinocandins showed that, although planktonic cells of *Candida* spp. were susceptible to all three echinocandins, the MICs of sessile cells were higher [63,64]. Even so, caspofungin and micafungin were active against *C. albicans* and *C. glabrata* biofilms, but not against *C. tropicalis* and *C. parapsilosis* biofilms [65,66]. Anidulafungin has also been tested alone and in combination with other antifungals and proved highly active against both planktonic and sessile cells, with planktonic and sessile MICs for 50% and 90% of the isolates tested (MIC_50_ and MIC_90_) of ≤0.03 µg/mL to <0.125 µg/mL and ≤0.03 µg/mL to ≤0.03 µg/mL, respectively [67,68]. When anidulafungin was compared with AmB, it displayed more activity than AmB against *C. albicans* biofilms after 24 h of maturation; however, AmB was more active against more mature biofilms (>48 h) [69]. Compared with azoles, anidulafungin had the lowest sessile MIC_90_ (0.063–0.125 µg/mL *vs.* ≥64 µg/mL), which was also maintained in long-term trials of continuous flow culture [70,71,72].

Similar results have been found in studies assessing the activity of micafungin against planktonic and sessile cells of *Candida* strains isolated from clinical samples: sessile MICs were higher than planktonic MICs in both *C. albicans* and *C. parapsilosis* [64,73].

However, in the case of antifungal lock therapy (ALT), high-dose echinocandins, especially caspofungin, had paradoxical effects on *Candida* biofilms [63,74,75,76]. Anidulafungin had the weakest *in vitro* paradoxical effect [77]. The clinical relevance of paradoxical effects remains unknown, although more and more data suggest that they might be overrated.

As for the inhibition of the metabolism of sessile cells, Cocuaud *et al.* found that caspofungin used at 2 µg/mL significantly decreased the metabolism of all of the strains of *C. albicans* and *C. parapsilosis* tested, independently of biofilm maturation [78]. A recent study by our group also demonstrated that concentrations of micafungin above 2 µg/mL were sufficient to inactivate re-growth of sessile *Candida* cells [64], as suggested in a previous study by Kaneko *et al.*, who analyzed real-time data comprising time-lapse images taken at brief intervals. Their results showed that, unlike fluconazole, micafungin contributed not only to fungicidal activity, but also to the immediate suppression of biofilm growth [79]. However, in an *in vitro* model of *Candida* biofilms on polystyrene and CVC sections, Seidler *et al.* found that micafungin could not reduce metabolic activity completely, even at the highest concentration [80].

Other *in vitro* studies have been carried out to increase the anti-biofilm effect of antifungals by administering them at high concentrations (ALT) in combination with systemic treatment. Toulet *et al.* found that AmB-L lock solutions (1000 µg/mL) strongly inhibited *Candida* spp. in young and mature biofilms for up to 48 h after the end of the lock period [81]. In their study of AmB and caspofungin, Oncü *et al.* reported similar findings, with a significant decrease in the *C. albicans* and *C. parapsilosis* colony count from baseline until the fifth day, when the catheters were completely sterile [82]. Other studies testing high doses of caspofungin and micafungin in silicone catheters also found reductions in the yeast metabolic activity of intermediate and mature biofilms (between 12 h and five days) [83,84]. In contrast, Ko *et al.* tested the *in vitro* activity of ALT against *C. albicans*, *C. glabrata* and *C. tropicalis* biofilms using five antifungal agents in polyurethane catheters and showed that only azoles were able to eliminate the viability of the biofilms of all *Candida* species within 7, 10 and 14 days [85].

New approaches are being developed to improve the effect of ALT by combining it with other non-antibiotic/antifungal agents. The addition of chelators, especially in combinations, is an innovative and superior alternative to heparin lock solution in the prevention and treatment of CRC. Cinnamon oil was effective against *C. parapsilosis* biofilms at 250 µg/mL, although it did not show a synergistic effect when combined with AmB [86]. Xanthorrhizol at 128 µg/mL exhibited potent activity against *C. glabrata*, *C. guilliermondii* and *C. parapsilosis* biofilms at the mature growth phase [87]. Percival *et al.* showed that using 40 mg/mL of tetrasodium EDTA in a lock solution for at least 21 h significantly reduced CVC-associated biofilms in a *C. albicans* model [88]. Rane *et al.* demonstrated that concentrations of 35% ethanol or higher for a minimum of 4 h reduced the metabolic activity of mature *C. albicans* biofilm by more than >99% and prevented re-growth [89]. Minocycline-EDTA-25% ethanol fully eradicated Gram-positive, Gram-negative and fungal biofilms within 2 h and completely prevented re-growth [90,91]. Moreover, a solution combining 0.01% glyceryl trinitrate with 7% citrate and 20% ethanol showed a synergistic effect in eradicating *C. albicans* biofilms in an *in vitro* model of catheter colonization [92].

### 4.2. In Vivo Activity of Antifungals Administered to Eradicate Candida Biofilms

Recent *in vivo* studies analyzed the efficacy of antifungals against *Candida* biofilms in both systemic and lock therapy. AmB-L was able to eradicate *C. albicans* biofilms formed on catheters placed in rabbits exposed to AmB-L lock therapy [93,94]. In contrast, when deoxycholate AmB (AmB-D) was compared to caspofungin in a model combining systemic and intraluminal lock therapy for seven days, caspofungin was more effective at eradicating *C. albicans* biofilms [95]. Moreover, caspofungin showed promising results when tested in lock therapy to prevent *C. albicans* biofilms in a murine model [96]. Kucharikova *et al.* found that all echinocandins were effective against *C. albicans* biofilms developed in a subcutaneous catheter rat model when administered intravenously for 5, 7 and 10 days [97]. Testing of anidulafungin against mature *C. albicans* biofilms in rats showed that seven-day systemic administration reduced cell numbers in catheters retrieved from treated animals compared with those retrieved from untreated and fluconazole-treated animals [98].

### 4.3. Clinical Studies on the Management of Candida Biofilm in CRC

#### 4.3.1. Therapeutic Approaches

International clinical practice guidelines for the management of candidiasis recommend withdrawing the catheter when there is suspicion of CRC [99]. However, this approach is problematic in the case of indwelling catheters, such as tunneled catheters or totally implantable venous access ports. Replacement of these catheters requires surgery and is expensive. Moreover, patients with these types of catheters (patients receiving parenteral nutrition, hemodialysis or chemotherapy) may not have any other available vascular access [100]. Therefore, some authors are considering the possibility of managing patients with CRC while the catheter remains in place [101,102,103]. Garnacho-Montero *et al.* evaluated the outcome of candidemic patients with CVC and found that delay in catheter withdrawal and in administration of adequate antifungal therapy was associated with increased mortality [104]. In contrast, Nucci *et al.* found that early removal of CVCs in non-neutropenic patients was of no clinical benefit when patients were covered with antifungals that were active against biofilm [101]. This finding was supported by other authors, who showed that outcome was better in patients with candidemia receiving highly active anti-biofilm agents [105]. It seems that that timing of CVC removal is best determined after carefully considering risks and benefits and that adequate anti-biofilm antifungal therapy is the key to better outcome [2,106].

Biofilm is not associated with increased mortality or development of CRC. Tortorano *et al.* found that 25.7% of *Candida* isolates from blood presented high biofilm-forming ability, but they did not find differences in the crude mortality rate according to this ability in *C. albicans* and non-albicans *Candida* (33.3% *vs.* 24.1%, OR = 1.57) [13]. A similar study performed recently by our group revealed no differences in mortality rate between patients colonized by biofilm-forming isolates and patients colonized by non-biofilm-forming isolates (24.4% *vs.* 22.2%, *p* = 0.776) and that biofilm production was not a good predictor of CRC [48].

The approaches developed in recent years to address management of CRC include catheter lock antiseptic solutions, antiseptic-coated catheters and Luer-activated needleless connectors have been optimized in recent years [88,89,91,92,107,108,109,110,111,112,113].

ALT is a novel therapeutic approach in catheter salvage that has been described in *in vitro* and *in vivo* studies. The procedure consists of filling the catheter lumen with high doses of antibiotic/antifungal solution alone or in combination with other components for a period of time during which systemic toxicity is avoided and serum drug levels are monitored. The recommended period for treating an episode of C-RBSI with combined systemic treatment, and ALT is 7–14 days after the last negative blood culture (instillation periods of between 2 h and 48 h) [114]. The success rate of ALT in catheter salvage described in C-RBSI and CRC is between 71.4% and 82% [115,116]. Most studies describing the outcome of patients receiving systemic treatment and ALT were performed with AmB-D and AmB-L, which were associated with a 60% salvage rate [117,118,119,120,121,122,123,124,125,126,127]. Blackwood *et al.* performed the only study with azoles. Three patients were successfully treated with systemic fluconazole or voriconazole in combination with ethanol lock therapy [128]. As for echinocandins, the literature contains only one report of a patient treated with systemic caspofungin in combination with ALT: the patient had *C. lipolytica* CRC that was treated successfully without removing the catheter [120]. Recently, five of seven cases of CRC (71.4%) were successfully treated with ethanol lock therapy and systemic echinocandins [115].

In extra-luminal biofilm, cells do not detach as easily from the biofilm; however, some clinical failures of ALT could be due to the presence of extra-luminal biofilm.

Even though most research articles based on clinical studies point out that micafungin is one the most active agents with anti-biofilm effects in adult and pediatric populations [129,130,131,132,133], future research is needed to evaluate its anti-biofilm effect in the management of CRC by combining systemic treatment and lock therapy.

#### 4.3.2. Novel Preventive Approaches

In order to improve the management of CRC caused by biofilm-forming *Candida*, preventive measures, such as antiseptic-coated catheters and Luer-activated needleless connectors, have been optimized in recent years. 

Privet *et al.* demonstrated that the combination of nitric-oxide with silver sulfadiazine had a synergistic effect against adhesion and biofilm formation in *C. albicans* [134]. Roe *et al.* showed that plastic catheters coated with bioactive silver nanoparticles had significant *in vitro* antimicrobial activity and provided 10 days’ protection against the formation of *C. albicans* biofilm [135]. Other authors found that the formation of *Candida* biofilm could be prevented with chlorhexidine-minocycline-rifampin-impregnated catheters alone and in combination with gendine [110,136]. These impregnated catheters have proven effective both *in vitro* and *in vivo*. Cobrado *et al.* showed that subinhibitory concentrations of cerium nitrate and chitosan significantly reduced biofilm formation by *C. albicans*, making both options promising alternatives for coating CVCs [109]. Martínez *et al.* also showed chitosan to be active against fungal biofilms at doses that were not toxic for human endothelial cells [113].

Impregnated catheters had been tested in clinical studies of adult critical care patients. Khare *et al.* demonstrated diminished catheter colonization using a silver zeolite-impregnated CVC [112], and Carrasco *et al.* showed that the use of CVCs coated with chlorhexidine and silver sulfadiazine reduced the risk of catheter colonization by *Candida* spp. [137]. Walz *et al.* showed that CVCs coated with 5-fluorouracil were also effective against colonization and could serve as an alternative to catheters externally coated with chlorhexidine and silver sulfadiazine [138].

Other novel preventive strategies, such us antiseptic barrier caps for needleless connectors, have proven effective in the prevention of contamination, both *in vitro* and in clinical studies [111,139,140,141].

**Table 1:** Summary.

***Candida* Biofilm**
*Candida* biofilm is produced by an initial attachment of cells coated with glycoproteins that progresses to a three-dimensional structure.
*C. albicans* and *C. parapsilosis* are two of the main biofilm-producing species.
The ability of these strains to produce biofilms may explain the high frequency of CRC.
Fungal biofilm on CVCs could act as a reservoir for re-infection and necessitate catheter withdrawal.
**Activity of antifungals against *Candida* biofilm**
Echinocandins are the most active agents against *Candida* biofilms in *in vitro* and *in vivo* models.
The MIC profiles of echinocandins in sessile cells are higher than in planktonic cells.
AmB-L had a good anti-biofilm effect when used at high doses.
In general, *in vitro* studies demonstrated that the anti-biofilm effect of antifungals increased when the drugs were used at high concentrations (ALT).
The addition of chelators to ALT provides an innovative and superior alternative to heparin lock solution in the prevention and treatment of CRC.
**Clinical aspects of *Candida* biofilms**
Adequate anti-biofilm antifungal therapy, rather than early CVC removal, is the key factor for a better outcome.
The optimal period for treating an episode of CRC with a combination of systemic agents and ALT is 7–14 days after the last negative blood culture.
The therapeutic success rate of ALT with catheter salvage in CRC is between 71.4% and 82%.
Most studies describing the outcome of patients receiving systemic treatment and ALT are performed with AmB-D and L-AmB, which were associated with a 60% catheter salvage rate.
In recent years, preventive measures, such as antiseptic coated catheters and Luer-activated needleless connectors, have been optimized to improve the management of CRC.

CRC, catheter-related candidemia; CVC, central venous catheter; L-AmB, liposomal amphotericin B; ALT, antifungal lock therapy; AmB-D, amphotericin B deoxycholate.

## 5. Conclusions

Numerous *in vitro* and *in vivo* models of CRC have demonstrated the effectiveness of antifungals, especially echinocandins, both alone and in combination with other components in the eradication of *Candida* biofilms (Table 1). However, more prospective studies should be performed to evaluate the clinical significance of these findings by combining systemic antifungals with ALT in patients with CRC for whom catheter removal could pose more of a risk than a benefit.
